# Concluding Commentary: The development of health professional educators

**DOI:** 10.15694/mep.2018.0000138.1

**Published:** 2018-06-27

**Authors:** Gary D. Rogers

**Affiliations:** 1 School of Medicine

**Keywords:** Faculty development, MedEdPublish special issue, Health professional educators

## Abstract

This article was migrated. The article was marked as recommended.

No abstract.

## Introduction

The eight original contributions to this themed issue of *AMEE MedEdPublish* span the breadth of the diverse enterprise aimed at enhancing the capabilities of health professional educators globally. They include the full range of methodologies, from narrative accounts of program implementation to formal qualitative, mixed methods, quantitative and ‘learning analytic’ studies, as well as a broad array of topic areas from skills development in veterinary practice to the promotion of professionalism in a medical school. What they all have in common is a focus on developing and supporting the academics and clinicians who educate health care workers to enhance their capabilities and improve their pedagogical effectiveness.

## Synthesis of themed issue content

Crichton and Jones (2018) reported a mixed methods study that sampled a large number of medical clinical supervisors in Scotland, following the introduction of a compulsory requirement to provide evidence of training in health professional education. They found that the imposition of this requirement was viewed negatively by many, even when respondents recognised the benefits of additional training in education. It left some feeling despondent, demotivated and considering withdrawing from participation in the education of junior doctors. They compared the opinions of supervisors who had enrolled in a formal ‘Clinical Educators Program’, provided by local universities and health services, with those who had not. They found that educators involved in the program felt less isolated and benefited from activity that appeared to have some of the functions of a community of practice ( Lave & Wenger, 1991). They also found that many respondents cited lack of time in the clinical environment as an important barrier to effective supervision, as well as to participation in activities to enhance educational quality.

Hassanien and Abou-Kamer (2018) reported on their utilisation of 19 YouTube video-based lectures on diverse topics to support the development of medical educators, primarily in Saudi Arabia and Egypt. They employed the ‘learning analytics’ data provided by the hosting platform to evaluate the learning method. The authors found that the videos were generally well-received by learners, with a high proportion of ‘likes’ and ‘shares’, as well as very few ‘dislikes’. They also reported that the length of the videos appeared to have a profound effect on ‘audience retention’, with shorter videos being much more likely to be viewed in their entirety.

Johnson and Williamson (2018) from the United States provided a narrative account of their faculty development work with clinical skills instructors at a veterinary college, specifically in relation to the introduction of new ‘low stress’ handling techniques for companion animals. They utilised a summer workshop for instructors, augmented by refresher sessions immediately prior to each student workshop, and found that this approach enabled instructors to acquire the new techniques effectively, facilitating the smooth introduction of the new skills content to student learning.

In a paper from Australia, Keir, Saad & Davin (2018) reported on interviews with 14 medically-qualified problem-based learning facilitators, at a single medical school, focused on student acquisition of diagnostic reasoning skills, with the aim of informing facilitator development in this area. They utilised a deductive and interpretative analytical methodology and found that, while facilitators often believed that explicit teaching strategies in relation to diagnostic reasoning should be employed, in reality they reported working mostly intuitively. The authors concluded that developmental programs needed to be developed to assist facilitators with the use of specific strategies to enhance student knowledge organisation and their understanding of metacognition.

Lazor, Takahashi and Leslie (2018), in a paper from Canada, explored the important nexus between faculty development and curriculum development in health professional education. Through collaborative reflection within their own team, and then at national and international conferences, they have developed a new practice model that integrates concomitant and complimentary faculty development at each stage of the curriculum development process. Their descriptive paper highlights the synergies that can be achieved when these two essential components of curriculum renewal are considered together.

In another paper from Canada, Miller and colleagues (2018) reported on three case studies of attempts to initiate communities of practice ( Lave & Wenger, 1991) to support the ongoing development of health professional educators at different sites. They utilised a grounded theory-based ( Charmaz, 2006), qualitative evaluation strategy and found that, despite concerted efforts by the practitioners at all three sites, none was successful at implementing and sustaining a vigorous community of practice. The group concluded that discontinuity resulting from cancelled meetings and incommensurable schedules (related to the pressure and complexity of contemporary academic life, incorporating clinical care, education and research) contributed to the lack of sustained success of the endeavours. They also postulated that a desire to span both teaching practice and educational scholarship may have led to difficulty in maintaining the common focus required for a community of practice to be perpetuated.

On a very different tack, Schlegel, McLeod and Selfridge (2018) from the United States recommended utilisation of the tools of formal project management to support the development and implementation of new learning activities, providing 12 ‘tips’ and a set of practical tools to assist educational designers to this end. They argued that the techniques of project management have much to offer health professional educators as they attempt to design and implement educational innovations.

Finally, Wadland and colleagues (2018), also from the United States, provided a detailed narrative account of the development of a framework and program to support professional behaviour among both students and faculty of a single medical college, through the shared conception of the ‘virtuous professional’. Under this approach, all members of the school’s learning community are viewed as professionals who aspire to be virtuous. Both student learning and faculty development are guided by a common statement of values that inform case-based learning activities, as well as appraisal and career development processes for educators.

## Take Home Messages

The diverse papers included in this themed edition of *AMEE MedEdPublish* underline the breadth and complexity of the processes undertaken globally in order to develop and support the capabilities that educators, across the full range of health professions, require to undertake their vital work effectively. Collectively, they argue for a focus of attention and resources on a thoughtful and concerted approach to this important area. Effective programs should encompass scholarly attention educators’ development needs, careful design and execution of faculty development programs informed by evidence and theory, then meticulous evaluation of these innovations, utilising the full range of investigative strategies and subsequent modification of the learning activities based on the findings.

## Notes On Contributors

Gary D. Rogers MBBS, MGPPsych, PhD, FAMEE, FANZAHPE, PFHEA is Professor of Medical Education Deputy Head (Learning & Teaching) of the School of Medicine, Griffith University, Queensland, Australia, as well as a role as Program Lead for Interprofessional and Simulation-Based Learning in the Griffith Health Institute for the Development of Education and Scholarship (Health IDEAS).

## Declarations

Professor Gary D. Rogers is the guest Theme Editor for the AMEE MedEdPublish theme - The development of health professional educators.
