# Supplementary material for: Concluding Commentary: The development of health professional educators
Source: MedEdPublish (2016). 2018 Jun 27;7:138. [Version 1] doi: 10.15694/mep.2018.0000138.1 (PMC10701847; doi:10.15694/mep.2018.0000138.1)
Supplement: Peer review history

# Reviewer report: Reviewer response for version 1

**DOI:** 10.21956/mep.19605.r29122

**Published:** 2019-02-24

**Authors:** Trevor Gibbs

**Affiliations:** 1 AMEE

This review has been migrated. The reviewer awarded 5 stars out of 5 An excellent paper that draws together several papers related to a very diverse topic.Resources and faculty development stand out from the papers, both very important but still require quite a lot of work to be done in their area. I would happily suggest that not only this paper but the papers in the theme should be read by all involved in curriculum development.

Reviewer Expertise:

NA

No decision status is available

# Reviewer report: Reviewer response for version 1

**DOI:** 10.21956/mep.19605.r29123

**Published:** 2018-07-05

**Authors:** Sateesh Babu Arja

**Affiliations:** 1 Avalon University School of Medicine

This review has been migrated. The reviewer awarded 5 stars out of 5 I thoroughly enjoyed reading this commentary. It is good to see the concluding commentary on the development of the health professional educators. I joined the AMEE MedEdpublsih forum in the middle of this theme. But this commentary helped me with the articles that were published on this theme. It is very important to see the faculty development activities for the health professional educators during the process of developing healthcare providers to be the effective teachers. I liked the article by Crichton and Jones (2018) based on a study conducted in Scotland. Clinicians perceived negatively regarding the clinical educators' program even though they recognized the benefits of the program. The other article that got my attention is Hassanien and Abou-Kamer (2018) in Saudi Arabia and Egypt. They clearly explained the role of youtube videos in faculty development activities and how short videos are more effective. The other paper that I was excited is the paper from Australia, Keir, Saad & Davin (2018). This paper is based on how faculty development activities for problem-based learning facilitators improved their diagnostic skills. Thank you for a wonderful commentary. These themed articles on faculty development are very useful for the faculty members and the deans and administrators who are monitoring the faculty development activities.

Reviewer Expertise:

NA

No decision status is available
